# Historical and current issues in HIV encephalitis, and the role of neuropathology in HIV disease: a pathological perspective

**DOI:** 10.1007/s00415-022-11503-2

**Published:** 2022-12-02

**Authors:** Sebastian Lucas

**Affiliations:** Guy’s & St Thomas’ NHS Foundation Trust, London, SE1 7EH UK

**Keywords:** HIV, Encephalitis, CD8, Pathology, Neurocognitive, Brain

## Abstract

In the 1980s, after the HIV pandemic was recognised, neuropathology identified cerebral white matter lesions that were found in the brains of infected persons with a severe irreversible dementia syndrome, this became known as ‘HIV encephalitis’. Subsequent work in Europe and north America found subtle morphological abnormalities in cerebral neurones and their connections. With the advent of effective anti-retroviral therapies after 1996, the incidence of severe HIV-related dementia declined, as did investigative tissue pathology into this HIV brain disease. Currently, the intense interest over HIV neurocognitive impairment focuses on neuroimaging, comparative blood and cerebrospinal fluid analysis, viral subtype analysis, and the search for biomarkers that correlate with brain function. Tissue neuropathology in HIV is more restricted to the diagnosis of acute disease such as opportunistic infections and tumours, and confirmation of the acute CD8 + T-cell encephalitis syndrome. But correlative tissue pathology will still be needed as newer therapeutic measures are developed to prevent and manage chronic HIV brain impairment.

## Introduction

Before the advent of effective anti-retroviral therapy (ART, from 1996), HIV brain and central nervous system (CNS) pathology seemed relatively straightforward diagnostically.

**Fig. 1:**
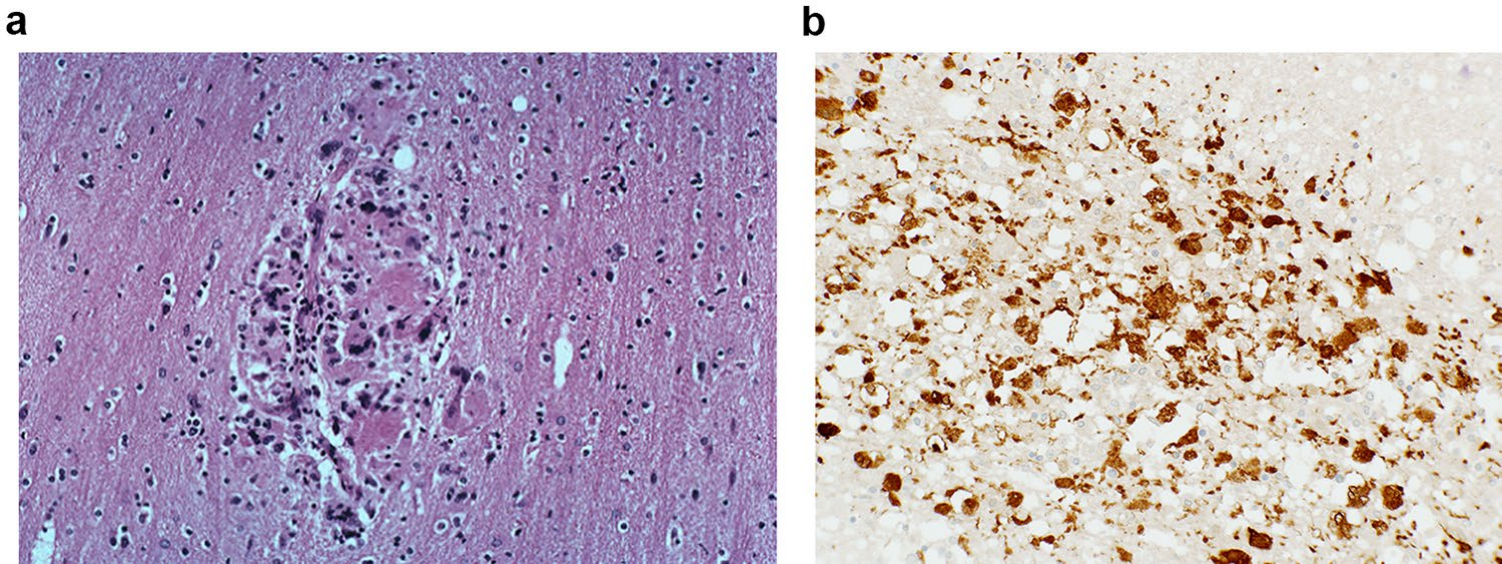
Classic HIV encephalitis. **a** H&E: Cluster of microglia and giant cells in the white matter. **b** Anti-HIVp24 immunostain, showing HIV virus within the microglia

There were very common immunodeficiency-related opportunistic infections and B-cell lymphomas, which neuropathology played a critical role in delineating and advertising, particularly from autopsy. There was a late HIV-related clinical dementia syndrome (HIV-associated dementia, HAD) where many patients had a histopathological *HIV encephalitis* (HIVE): multiple white matter foci of multinucleate microglial giant cells (MGC), containing demonstrable HIV virus using immunohistochemistry or in-situ hybridisation, ± microglial nodules (MGN) [1]; this was a new, unique pathology (Fig. 1). There was also a variant of HIVE termed *HIV leukoencephalopathy* (HIVLE), characterised by diffuse white matter damage with prominent myelin loss and astrocytosis, alongside the MGC, MGN and demonstrable HIV virus.

Underlying cerebrovascular disease (haemorrhage and infarction) was there and aggravated by HIV infection [2]. In that pre-ART era, nearly all presenting HIV + ve patients died of AIDS within a decade from infection, and there was a high autopsy rate with much CNS pathology.

As well as infections, stroke and HIVE, there were numerous case reports of more difficult to comprehend entities: e.g. acute disseminated encephalomyelitis (ADEM), acute relapsing brain oedema, fulminant multiple sclerosis-like leuko-encephalopathy, tumefactive demyelination, acute fulminating fatal leukoencephalopathy, occipital posterior reversible leukoencephalopathy syndrome (PRES), focal pontine leukoencephalopathy, rapidly progressive degeneration of auditory, visual and corticospinal tracts. At the time, these were pathogenetically unexplained, and in our current era of ART, similar clinical pathologies still present, and we still puzzle as to whether or not they are directly caused by HIV infection, or are a coincidental process, and how to categorise them [3]. Antibody-driven autoimmune encephalitis syndromes are occasionally described in HIV-infected patients at various stages of their infection; how they pathogenetically relate to HIV is also unclear.

Four things changed from 1996. First, effective ART and its roll-out improved progressively, with the current expectation of a nearer normal life span, without a classical AIDS-defining condition developing, as long as the HIV infection is caught early and patients take the ART. As a corollary, the severest HIV dementia syndrome, HAD, and the classic HIVE pathology became uncommon [4]. Secondly, CNS imaging developed in its range of modalities and apparent ability to identify underlying pathology in the CNS, rendering brain biopsy as a diagnostic tool less common. Thirdly, the clinical science of HIV-associated *neurocognitive impairment* (NCI) took off with more complex assessment tools and increasingly sophisticated studies of cerebrospinal fluid (CSF), whilst the tissue neuropathology to explain NCI advanced slowly. And fourthly, from around 2002, it became evident that some patients with apparently well-controlled HIV infection unexpectedly deteriorated, and many died from cerebral swelling, associated with a florid *CD8* + *T-cell encephalitis* (HIV-CD8E) [5]. Some of these patients had the ART-related syndrome of immune reconstitution inflammatory syndrome (IRIS) in the brain.

Consequently, what is currently meant by ‘HIV encephalitis’ is complicated. Should it be restricted to the original 1991 histopathological case definition (hereon labelled ‘classic HIVE’)? Or, more broadly, does HIVE apply to all inflammatory brain syndromes in people, with proven HIV infection, and should it include or exclude conditions attributable to other infections, stroke, and vasculitic disease? Does it include the seroconversion, self-limiting acute meningoencephalitis syndrome? Does the diagnostic label ‘HIV encephalitis’ require supportive tissue pathology, or is imaging and CSF/blood analysis sufficient? Further, is an HIVE the underlying basis for most or all the neurocognitive impairment syndromes?

A PubMed search for ‘HIV encephalitis’ finds > 6000 items published between 1983 and 2022, the peak year being 1995 (pre-ART). A search for ‘HIV encephalitis’ on Yahoo brings up an online article that explicitly opens: “HIV encephalitis, also referred to as HIV-associated neurological disorder (HAND), includes a range of neurocognitive defects of varying severity following HIV infection” [6]. This would suggest that we abandon neuropathological purity and open up ‘HIV encephalitis’ to any and all central nervous syndromes caused directly or indirectly by the HIV virus.

This review concerns how we think about HIV encephalitis and, specifically, what role neuropathology currently has in its definitions and pathogenesis. The focus is on adults. (Paediatric HIV/AIDS brain disease is complicated by (a) the fact of brain growth and development co-existing with brain HIV infection, and (b) the relative infrequency of paediatric brain neuropathology examinations—historically, let alone now—compared with those of adults; and so is not discussed further.) It is taken for granted that brain biopsy and autopsy follow-up remain important clinically for identifying opportunistic infections, lymphomas and vascular disorders, whilst noting that the number of such investigations has significantly declined over the last two decades (discussed more below). The main focus is to consider through *tissue neuropathology* how HIV causes neurological diseases, both historically and in the current and future care of patients.

A global perspective is needed for context. By 2020, 38 million people worldwide were living with HIV, of whom two-thirds receive ART. The burden of HIV-associated debilitating neurocognitive disorder (NCD) is not precisely known. The original 2007 criteria for diagnosing the 3 stages of HIV-associated neurocognitive disorder (HAND)—asymptomatic (AND), mild (MND) and HIV-associated dementia (HAD)—included clinical assessment and psychomotor tests [7]. Recently there is realisation that the NCDs are probably overdiagnosed through including non-specific psychomotor metrics [8]. Nonetheless the burden is huge, with an estimated 16 million cases of HAND, in both ART-treated and ART-naïve patients; and an estimated 72% of these patients live in sub-Saharan Africa [9]. The clinical and pathological investigation of HAND is, however, almost entirely undertaken in middle and high income countries outside Africa [10]. The only systematic study of HIV-related neuropathology in sub-Saharan Africa was undertaken three decades ago, before the introduction of ART. In patients dying of advanced HIV disease, we found much CNS opportunistic infection, little cerebral lymphoma, and < 1% frequency of classic HIVE brain disease [11], contrasting with the 10–25% prevalence of classic HIVE in high-income countries at that time [12].

## Three phases of HIV neuropathology investigation

By the time of coining the term ‘AIDS dementia complex’ (1986), the main associated histological features were known: microglial nodules and multinucleate giant cells containing HIV virus, predominantly in the white matter rather than cerebral cortex. And the mode of entry of HIV into the brain within infected monocyte/macrophages, across the blood–brain barrier, early in infection, had been demonstrated. By 1987, the debate over the pathogenesis of these encephalitic lesions commenced: whether due to a toxic effect of HIV (e.g. Tat protein) on neurones and their connections, or an indirect effect of secretions from HIV-infected microglia/macrophages.

There have been three distinct phases of brain clinical pathology research focussed on the chronic condition of HIV-related neurocognitive impairment. First, the decade following 1981, after the official recognition of what we now term HIV/AIDS, autopsy brain examinations in USA and Europe which resulted in the 1991 consensus definitions of classic HIV encephalitis (Table 1) [1].

**Table 1:** The original primary HIV CNS pathologies [1]

HIV-specific CNS pathologies	HIV-associated CNS pathologies^a^
HIV encephalitis [HIVE]	Spinal cord vacuolar myelopathy
HIV leukoencephalopathy [HIVLE]	Diffuse poliodystrophy
HIV meningitis	Spongiform encephalopathy

^a^i.e. similar pathology can be seen in non-HIV patients

There followed the second phase from 1991, a period of extraordinarily detailed neuropathological investigation into the damage to neurones and their connections in HIVE in the UK and USA, seeking to understand the pathogenesis of the HIV dementia syndrome—HAD [12].

By studying neurones and their connections, attention shifted from the mainly sub-cortical (white matter) lesions of classic HIVE to the probably more critical effects on nerve cells. The density of frontal cortical neurones was shown to be significantly reduced in people with HIV compared with non-infected controls; confocal microscopy revealed dendritic injury, whilst 3-dimensional stereology illustrated reduced synaptic densities. It is significant that across all these studies, the severity of such abnormalities correlated poorly with the amount of classic subcortical HIVE lesions, and with pre-mortem assessments of neurocognitive impairment.

The possible role of co-morbidities contributing to neurocognitive impairment began to be realised, some more common in MSM patients or illicit drug users than in heterosexual patients, often relating to age and life style. The long list includes cerebral artery atherosclerosis, smoking, alcohol and other substance abuse direct toxicities, hepatitis B and C infection, direct head trauma, and the deposition in the brain of neurodegenerative proteins usually associated with Alzheimer disease such as tau and amyloid beta, and there is the ageing process itself [4, 13].

By 1996, the pathology of early HIV infection had been documented serendipitously (patients at this stage of HIV disease died mainly from accidents and substance abuse, thus requiring medicolegal autopsies). At the HIV seroconversion stage, the brain showed a mild T-cell lymphocytic meningoencephalitis, no classic HIVE, and no identifiable HIV virus with immunohistochemistry [14].

Towards the end of this phase, the golden age of HIV neuropathology [12, 15], in the high-income countries where HIV neuropathology was undertaken, the number of patients developing HAD had dropped dramatically. The treatment for the condition, as well as its prevention, was obviously ART, and to be realistic, little more had been learned from the histo-morphological studies that could be transferred directly and therapeutically to people living with HIV that would enable them to live better lives. In ART-treated patients, classic HIVE was no longer seen in brain material, replaced by perivascular chronic inflammation, focal white matter gliosis and neuronal atrophy—all non-specific, possibly ‘burnt-out’ lesions [13].

A review summarising the inconsistent correlation of neuropathology and clinical HAND, suggested that we think in terms of the ‘neurovascular unit (NVU)’. This is the blood vessel compartment in the CNS and its interface with a web of physical and functional interactions with brain parenchyma, including astrocyte foot processes, neurones and synapses, endothelial cells and pericytes, perivascular macrophages, microglia, CNS extracellular spaces, subarachnoid space and CSF, and constituents of blood plasma [15]. The latter arises from the established observations of systemic inflammatory activation products in blood, derived from microbial translocation out of the gut, the intestinal cell mediated immunity system having been irrevocably damaged during the early phase of HIV infection in all people living with HIV (PLWH) [16]. Some HIV researchers (but not all) attribute many chronic HIV-associated syndromes to this subtle systemic inflammatory syndrome, including pulmonary hypertension, chronic obstructive pulmonary disease, ischaemic heart disease, liver fibrosis, venous thromboembolism, frailty, osteopenia, wasting, stroke and neurocognitive disorders. The advantage of the NVU approach is that it reminds us of the potential of the many co-morbidities in PLWH that can affect the brain, and that observable CNS dysfunction is not necessarily attributable to HIV alone: it might be a legacy of earlier co-morbidities. An implication is that the perturbation of the NVU may be important in patients who are virally suppressed through ART, whilst brain inflammation is more important in those without viral suppression.

The third phase of HIV brain clinical pathology research emerged with the delineation of the common but less severe neurocognitive disorders (NCD), using clinical and psychometric tests, coinciding with more systematic investigations using neuroimaging and an increasing range of blood and CSF tests for not only HIV virus, but inflammatory biomarkers of NCD [17]. What is striking to a pathologist is how classical neuropathology, the microscopic histopathology of the brain, hardly features in this latest research. Neuro-histopathology now is mainly concerned with identifying acute clinical conditions, via both biopsy and autopsy. HIV brain banks are a thing of the past. A recent publication states “a separate definition [from cognitive test performance] should be developed for *HIV brain pathology* [my emphasis] applicable to research and clinical settings, pivoting on neuroimaging findings, biomarkers, trajectory of symptoms, and/or demonstrated decline in cognitive test performance in relation of acquisition of HIV”. This illustrates how non-tissue brain pathology, i.e. chemical analysis, virology, and immunology, have taken over as the major pathological axes of investigation [8].

Effective as ART is in most patients with HIV, the virus is never eliminated. It resides, latent, in many organ sides including the brain; and it always re-emerges if and when ART is stopped, or ART drug resistance develops. However, in the brains of those well-controlled on ART, HIV itself is not visible with usual tissue microscopy and immunohistochemistry. But we know it is there from PCR analyses, and presumably even this small amount of virus can have a significant immunological and signalling effect on the surrounding brain tissues.

## HIV-CD8 encephalitis syndrome

**Fig. 2:**
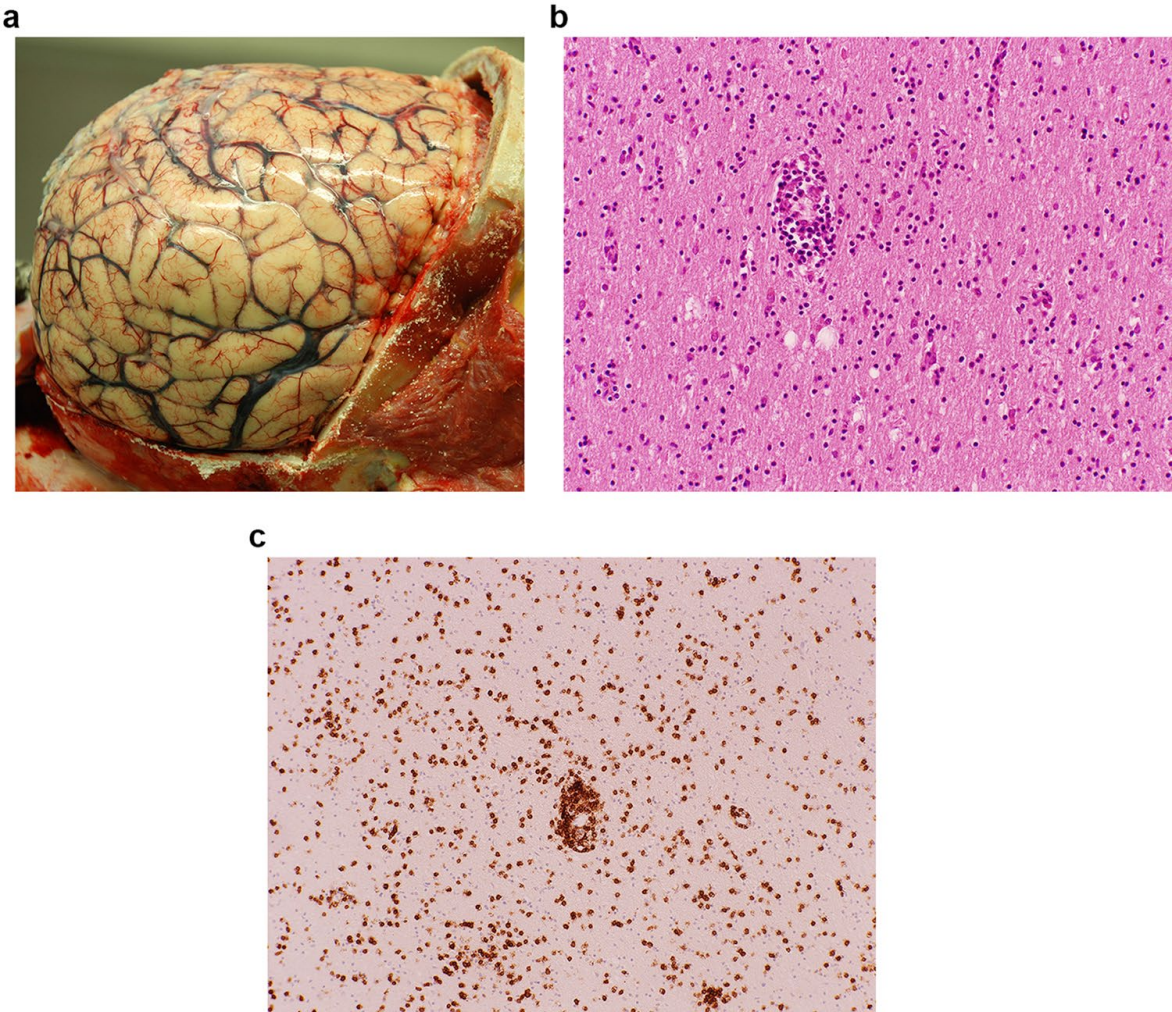
HIV-CD8 encephalitis. **a** Brain showing diffuse cerebral swelling. **b** H&E stain: abundant lymphocytes in the white matter. **c** Anti-CD8 immunostain, demonstrating their CD8 + T-cell lineage

One unambiguous HIV encephalitis syndrome that has emerged in the post-ART era is HIV-CD8 encephalitis (HIV-CD8E) [5]. Early cases developed in PLWH, on ART and well-controlled (i.e. blood viral loads undetectable), who became acutely or subacutely demented, sometimes starting with a seizure, with progression to coma and death in many cases. Imaging shows diffuse white matter changes and cerebral swelling/oedema. A definitive diagnosis is made on biopsy or at autopsy, with a diffuse and perivascular white matter infiltrate of CD8 + T-cells seen microscopically. Classic HIVE is not present, and nor is immunohistochemically demonstrable HIV; arterial and opportunistic infection pathology is excluded by definition, as is lymphoproliferative disorder (Fig. 2).

Treatment with steroids reverses the syndrome and has saved many lives, along with modification of ART regime, and so HIV-CD8E is now clinically suspected at an early stage and treated empirically after preliminary investigations. It is striking that Black people constitute the great proportion of patients (that is, Black PLWH in Europe and north America, since the syndrome has yet to be described in Africa, although it must be happening, undiagnosed as the investigative resources including neuropathology are not available). The Black predominance suggests a genetic contribution in pathogenesis.

Since the original depiction in 2004, the pathogenetic pathways to HIV-CD8E have broadened (Table 2), with interruption of ART the most common. CSF viral escape (see below), where data are available, is reported in two-thirds of all cases (however, the numbers are small). In the IRIS category there are not enough reports to draw conclusions about escape; and CD4 + T-cells are seen in addition to CD8 + T-cells. Beyond observing obvious perturbations in brain immunology and the blood–brain barrier in the six risk categories (Table 2), it is not yet clear why this syndrome happens and whether there is a mechanistic final common pathway.

**Table 2:** The risk categories for developing HIV-CD8 encephalitis [5]

1. Interruption of anti-retroviral therapy (ART)
2. Intercurrent infection or visceral malignancy
3. Immune reconstitution inflammatory syndrome (IRIS) after commencing ART
4. ART-drug resistance
5. Well-controlled on ART– no evident risk factor
6. Never received ART

## The decline of HIV neuropathology

Those of us who have, in the era of ART, been routinely examining autopsy brains from people dying of, and increasingly just with, HIV infection, have sought in vain to identify any consistent lesions that might correlate with non-catastrophic clinical neurological disorders. Neurone counting is not done now (it is very technical, with long processing times and time-consuming microscopy procedures) [12], and as all histopathologists know, just eye-balling cells, including neurones and glial cells, down the microscope is not going to inform on significant numerical differences unless the true numbers of cells being estimated differ by a logarithm or more. Staining astrocytes with GFAP, and microglia/macrophages with CD68, to estimate numbers and ‘activation’ is also subjective. Classical histopathology is relatively insensitive to quantitative changes in cells.

There is another reason for the decline of neuropathology in HIV-related NCD research: the decline in the number of pathologists with significant experience of HIV clinical pathology. Following the advent of ART, it is no longer the business it was, when HIV/AIDS was new and HAND had become a serious common problem to be investigated. Deaths from HIV/AIDS have declined in rich countries, and in many poorer regions thanks to the roll-out of ART [9], and so autopsy experience of HIV brains has declined. Most neuropathologists now experience only the occasional diagnostic problem in a biopsy from an HIV + ve patient. More importantly, they never experienced the pre-ART era, and thus have less confidence in identifying the HIV-related opportunistic infections, let alone classic HIVE and the occasional unclassifiable inflammatory pathologies that turn up (e.g. whether an unambiguous lymphocytic encephalitis is HIV-CD8E and/or IRIS reaction or not). At the moment, this might not matter so much. There are only limited therapeutic options in managing HIV CNS disease currently: varying ART regimes, immunosuppressive treatments such as steroids, and specific antibiotics against identified infections.

## The modern neuropathology of HIV-associated neurocognitive disorders

A new HIV CNS research industry has emerged. The focus is on examining CSF and blood fluids in parallel, studying HIV virus levels and cell tropisms, drug resistance, comparing CSF and blood lymphocyte counts, looking for biomarkers of brain inflammation, comparing different ART regimes, testing for compartmentalisation of HIV in the brain and viral escape, and all correlated with neuroimaging. The goal is to identify predictive biomarkers for NCD in CSF and, especially, in peripheral blood samples, and many patients have had been sampled sequentially over long periods of time [17]. This is evidently appropriate, since brain *tissue* sampling is increasingly difficult to include in research studies, for practical and ethical reasons. In the clinical management of HIV brain disease, biopsy is resorted to when imaging and CSF/blood analysis has not indicated a therapy, and occasionally to study suspected HIV resistance mutations, when it has not proven possible to obtain enough relevant sample from the CSF.

For an exhaustive review of the results of this latest phase of HIV brain research, a recently published review is highly commended [17]. Conceptually it follows from the older pre-ART classic neuropathology and then later morphological studies quantifying HIV quantities and numbers of macrophages and microglia, and correlating these with pre-mortem dementia. Virus was identified in both demented and non-demented patient brains, but some demented patients had no demonstrable virus. Macrophage and microglial quantitation correlated much better with dementia. The finding of brain virus in most patients dying of AIDS indicated, at the time of study in the 1990s, that it is not specific for HAD, whereas activated macrophage/microglia are more predictive. In this respect, ‘HIV encephalitis describes a pathological state that is not correlated with a clinical syndrome, unlike other viral encephalitides such as rabies, polio and herpes where the brain viral burden defines the clinical disease [18].

In the modern era of ART, when PLWH can still develop NCD despite ART, HIV virus is not routinely found in brain macrophages/microglia using standard histopathology and multinucleate giant cells are not found, we have less published neuropathology data. There are a few studies of patients on ART with progressive neurological dysfunction who have been biopsied, and who also have CSF viral escape. The biopsies find mixtures of mild CD8 + T-cell infiltrates and microglial activation [5, 19]; immunohistochemically demonstrated HIV virus is mostly not mentioned and is presumed not present. Importantly, the patients improved on changing the ART regimes.

CSF HIV escape (first highlighted in 2012) [19] is the replication of HIV in the CNS despite systemic viral suppression with ART: CSF viral load is greater than that in contemporaneous blood, or is detectable when the blood viral load is undetectable. CSF escape can be neurologically symptomatic or asymptomatic, and is associated with higher CSF levels of biomarkers such as neopterin. It is likely that CSF escape results from reactivation of latent HIV in the CNS long-lived macrophages and microglia and/or from HIV infection in the periphery into the brain (like a primary HIV infection).

Around the same time, the concept of compartmentalisation of HIV in the brain emerged. It is the evolution of CSF viral strains distinct from those found in the blood. Implicitly, virus in the CSF is the same strain as that in the brain neuropil, and it is suggested that such evolution takes place in long-lived brain macrophages and microglia [16]. Moreover, it possible that compartmentalised strains are maintained in the presence of ART and that these strains might re-emerge to infect blood cells if ART is discontinued.

In summary, the recent developments focus on brain disease that is attributable to HIV rather than to co-morbid factors, whether or not there is systemic HIV suppression, and whether there is CSF viral escape. The little descriptive pathology available of milder NCD events associated with viral escape, macrophage/microglial activation and discernible T-cell infiltration, i.e. an encephalitis, but no demonstrable HIV comparable with studies in the 1990s [18] shows overlap with the more florid CD8 encephalitis syndrome. The latter patients have much more CD8 + T-cell infiltration, but essentially there appears to be a continuum of severity of encephalitis from MND through to HIV-CD8E. The suggestion, back in 2013, that HIV-CD8E is ‘the tip of the iceberg’ of HAND is very reasonable [20].

## Biotypes of HAND: a summation approach

A new look at how PLWH develop NCDs, whether or not they are on ART, emphasises how patients with similar clinical phenotypes may have very different underlying disease processes, thus requiring different treatments. The proposition of different ‘biotypes’ of HAND is built on the older neuropathology but its case definitions mainly eschew diagnostic tissue pathology and rely on CSF and blood profiles of immunity, virology, and neuroimaging [21]. There are four biotypes proposed:

1. Macrophage-mediated HIV encephalitis: essentially the original classic HIVE in ART-untreated patients;
2. CNS viral escape, in ART-treated patients, with a lymphocytic encephalitis, elevated HIV viral load in CSF (viral escape), white matter hyperintensities on neuroimaging, and related to poor CNS ART penetration and ART drug resistance;
3. T-cell mediated encephalitis: associated with immune reconstitution reaction (IRIS) on ART, the target being HIV or opportunistic infection; pathologically there are CD4 + or CD8 + T-cell infiltrates in brain parenchyma (i.e. including CD8 encephalitis syndrome);
4. HIV-protein-associated encephalopathy: in ART-treated patients, with slowly progressive cognitive and psychomotor deterioration; pathologically characterised by neuronal loss, microglial and astrocyte activation with lymphocytic neuroinflammation; viral proteins detectable in CSF; also deposition of tau and Aβ in the brain.

The different therapeutic managements of these processes require a broad strategic diagnostic work-up. Pathologically, there is much to commend in this approach, although there is overlap of #3 and #4, as the CD8E syndrome is a morphological phenotype that crosses both categories. If the usage of these biotypes results in more efficient investigation and treatment of patients with less recourse to diagnostic brain biopsy than is current, then that is progress.

## Neuroimaging futures

In NCI imaging, two particular new methodologies may be able to investigate neuroinflammation and impacts of HIV on fine brain structure non-invasively. As discussed, a feature of NCI and chronic HIV brain disease is microglial cell activation. Positron emission tomography (PET) radiotracers can image translocator protein 18 kDa (TSPO) on activated microglia. Comparative studies, people living with HIV (PLWH) vs HIV-ve controls, and cognitively impaired vs unimpaired PLWH, have produced conflicting results although the overall picture is of detectable microglial activation in the cerebral cortex in the expected populations [22].

A second technology that has promise is the identification of reduced synaptic density in the brains of PLWH, using PET scanning with the SV2A ligand, correlated with T-1 weighed MRI images [23] supporting the morphological tissue observations of more than 20 years ago.

The application of these and future advanced technologies has two important caveats: (a) The extent to which the differences between the studied populations are the result of co-morbidities (as listed above) and medication side-effects, and (b) Whether the proposed pathogenic processes can be validated against parallel consistent alterations of the actual cellular and related constituents of brain tissues; i.e. do they correlate with morphology? It is not clear whether these goals are to be achieved, particularly whether histomorphology can ever be sufficiently granular for agreed interpretations and case definitions. Perhaps we might need those HIV brain banks back.

## Therapeutic futures

New immunomodulatory treatments, and preventions, for HIV-related neurocognitive disorders will surely come, supplementing the basic anti-retroviral therapies which are the bedrock of all HIV management. We expect to see the introduction of newer ART (with nanotechnology?) and immunomodulatory regimes, and perhaps gene-editing to eliminate HIV reservoirs, emerging from the NCD research [17].

A note of caution has been raised concerning new approaches to reversing HIV latency in tissues, particularly in the brain: the so-called ‘shock and kill’ method [24]. This provokes HIV from a latent state to an active state, more readily targeted by the immune system and ART. It is theoretically possible that this could precipitate a CD8 encephalitis syndrome [5].

So it would be wise to maintain some baseline HIV neuropathological experience as a form of quality control. There is no room here to summarise the large amount of experimental animal and cell culture work on HIV CNS pathology undertaken over the last two decades, but we expect useful insights into the neurological syndromes, their prevention, and treatment from such studies [25]

## Conclusion

Early in the AIDS pandemic, when the prospect of unlimited numbers of HIV-infected people developing untreatable dementia seemed all too real, great effort went into understanding the brain pathomorphology of the syndrome. ‘HIV encephalitis’ emerged as the substrate for many but not all cases, and we understood better the subtle changes in neurone numbers and connectivity in HIV-infected brains. The arrival of effective antiretroviral therapy put a stop to that work, as dementia incidence declined dramatically.

The neuropathology of most NCD now is mild lymphocytic infiltration plus macrophage/microglial activation that may or may not be detectable by usual microscopy. Tissue biopsy pathology will be restricted to the diagnosis of acute presenting syndromes in HIV, in the expectation of identifying infections, tumours, vasculitis and other inflammatory lesions. One significant role in HIV-related brain disease will continue: the diagnosis of suspected HIV-CD8 encephalitis, where it may be unclear whether there is an alternative pathology, such as lymphoma, toxoplasmosis or JC virus. CD8 encephalitis histopathology is characteristic, readily identified, and the effective life-saving treatment for the syndrome is well known.

So where does the term ‘HIV-encephalitis, HIVE’ sit now? My personal view is that patients who are demonstrated to fulfil histologically the 1991 case definition of HIVE, should be labelled as having *classic HIVE*. Generally, the 2010 consensus definition of an encephalitis should be applied: ‘the presence of non- pyogenic inflammatory infiltrates, commonly T-lymphocytes and microglia, within the brain’ [26]. HIV-CD8 encephalitis is a special case of HIVE given its distinctive clinical, radiological and histopathological features, but the term should be restricted to those cases with histological proof; suspected cases of CD8E, treated appropriately without recourse to biopsy (and hopefully death and autopsy avoided) should be termed ‘suspected/probable CD8E’. Where patients with evident neuroinflammation as evidenced from CSF samples, biomarkers and (in the future) neuroimaging, fit in is uncertain. I suspect that, tissue pathology purists to the contrary, they will be called ‘HIV encephalitis’ also.

## Data Availability

All data generated in this study are available in this article and the online supplementary material.

## Glossary of terms in the HIV brain disease

ADEM: Acute demyelinating encephalomyelitis
AIDS: Acquired immunodeficiency syndrome
AND: Asymptomatic neurocognitive disorder
ART: Anti-retroviral therapy, usually a combination of 2 or more drugs
CSF: Cerebrospinal fluid
CNS: Central nervous system
HAD: HIV-associated dementia
HAND: HIV-associated neurocognitive disorder
HIV: Human immunodeficiency virus
HIVE: HIV encephalitis
HIVLE: HIV leukoencephalopathy
HIV-CD8E: HIV-CD8 encephalitis, an acute or subacute encephalitic syndrome of global cerebral dysfunction, with white matter CD8 + T-cell infiltration
IRIS: Immune reconstitution inflammatory syndrome, a subacute syndrome developing after the initiation of ART in many HIV-infected patients
MGC: Multinucleate giant cell cluster
MGN: Microglial nodule
MND: Mild neurocognitive disorder
MSM: Men who have sex with men
NCD: Neurocognitive disorder
NCI: Neurocognitive impairment
NVU: Neurovascular unit
PLWH: Persons living with HIV
PET: Positron emission tomography
PRES: Posterior reversible encephalopathy syndrome
TSPO: Translocator protein
